# Neurological Abnormalities in Chinese Schizophrenic Patients

**DOI:** 10.1155/2007/451703

**Published:** 2007-08-22

**Authors:** Raymond C. K. Chan, Eric Y. H. Chen

**Affiliations:** ^1^Neuropsychology and Applied Cognitive Neuroscience LaboratoryInstitute of PsychologyChinese Academy of SciencesBeijingChina; ^2^Department of Psychiatrythe University of Hong KongHong Kong Special Administrative RegionChina

**Keywords:** Neurological signs, prevalence, Cambridge Neurological Inventory, schizophrenia, Chinese

## Abstract

*Background:* This study attempted to examine the prevalence and type of neurological signs in Chinese patients with schizophrenia.

*Methods:* A cross-sectional design was adopted with the use of the Cambridge Neurological Inventory (CNI). The CNI is comprised of 7 subscales, including motor coordination, sensory integration, disinhibition, extrapyramidal signs, dyskinesia, catatonia, and pyramidal signs. The former 3 subscales were classified as soft signs, whereas the latter 4 subscales were classified as hard signs. A total of 250 Chinese schizophrenic patients and 90 normal controls were recruited.

*Results:* Patients exhibited significantly more signs than normal controls in all subscales but pyramidal signs (p < 0.00005). Significant differences were also found in total soft signs, total hard signs as well as total neurological signs (p < 0.0005). The three subscales of soft signs showed a relatively better sensitivity and specificity as compared with the four subscales of hard signs. Improvement in sensitivity and specificity was demonstrated when the subscales were collapsed into total soft signs, total hard signs and total neurological signs. A cut-off of 4 in total soft signs yields a sensitivity of 0.63 and specificity of 0.71; whereas a cut-off of 1 in total hard signs yields a sensitivity of 0.78 and specificity of 0.89. A global cut-off of 5 in total neurological signs results in a sensitivity of 0.81 and specificity of 0.73 for detecting schizophrenia versus normal.

*Conclusions:* High levels of neurological abnormality characterize schizophrenic patients. An extended assessment battery of CNI provides even better discrimination of patients from normal controls, and soft signs are more strongly associated with schizophrenia than are hard signs in the Chinese sample.

## 1. Introduction

Schizophrenia is a major psychiatric illness affecting approximately 1% of the world population. It is an illness characterized by profound disturbances in perception, language, cognition, emotion, social functioning as well as neurological abnormalities. Neurological signs have been consistently shown to be present from early course of the illness [28,29] and do not seem to be secondary to neuroleptic medications [2]. In general, neurological abnormalities or signs are increased in schizophrenia as compared to nonpsychotic siblings, healthy subjects as well as other psychiatric disorders [4,14,18–20,32].

The crucial role of neurological abnormalities or signs in schizophrenia has been recognized by Tsuang and colleagues [35,36] as the “target features” that encompass the idea that genetic and non-genetic processes lead to maldevelopment in neurocognitive systems. Target features should be increased in relatives of patients but perhaps not to a similar extent. In addition, the manifestation of multiple genes of small effect would lead to an expectation that target features should be present, to a lesser extent, in the general population.

ISSN 0953-4180/07/$17.00 © 2007 – IOS Press and the authors. All rights reserved

Neurological signs therefore also represent a potential intermediate phenotype in schizophrenia [13].

Research on neurological signs in schizophrenia has provided strong evidence supporting the conceptualization of these signs as a trait feature. The study of potential relationships with symptomatology has shown negative [12,30,34,39] and disorganized [22,30,34] symptoms to potentially be significantly related to neurological impairment, especially prefrontal/frontal and parietal signs [22,30,34], whereas positive symptoms appear to be unrelated to neurological soft signs. Bombin et al. [6] have suggested that the lack of a significant soft signs-positive symptom relationship is expected, since neurological impairment is hypothesized to be a trait feature, whereas positive symptoms are state dependent. However, negative symptoms tend to be more stable across the course of the illness, and their presence may therefore predate the diagnosis of schizophrenia.

However, there are still a number of issues that are not well understood and need to be further examined. Firstly, definition and the corresponding assessment procedure vary across different studies. Neurological abnormalities or signs have previously been defined as "hard" and "soft" signs. Hard signs refer to impairments in basic motor and sensory behaviour such as signs for the "pyramidal" [40] and "extrapyramidal" systems [27,31,33], whereas soft signs refer to nonlocalizing neurological abnormalities that cannot be related to impairment of a specific brain region or are not believed to be part of a well-defined neurological syndrome [11,18]. However, this distinction may be artificial and may reflect an inability to define the brain-behaviour relationship that underlies the presence of neurological soft signs [5,18]. The ambiguity in characterizing the distinction between neurological soft and hard signs has led to differences in the categorization of neurological signs in the instruments used for the evaluation of these signs. The recent development of neuroimaging techniques may be useful in quantifying and differentiating the meticulous differences (e.g. [3]).

However, the use of rating scales still reserves their importance in clinical practice because of their portability, time-savings, and impressive psychometric properties. Clinicians should appreciate and acknowledge the differences of categorization of neurological signs, neurological soft signs in particular. For instance, Heinrichs & Buchanan [18] classified "soft signs" into three categories, namely "sensory integration", "motor coordination", and "sequencing of complex action"; whereas Chen et al. [11] operationally defined them into "motor coordination", "sensory integration", and "disinhibition". While the first two categories of the different classifications share common nomenclatures, the items included in motor coordination differ from each other to a certain extent. In Heinrichs and Buchanan's [18] classification, the boundary of items between complex motor acts and motor coordination has not been thoroughly considered. Examination of the individual items shows that, similar to the complex sequencing subgroup, a number of signs in the motor coordination subgroup also involve repetitive alternation in hand positions (e.g., finger thumb opposition, and diadochokineses). The difference between these and Luria's signs [21] appears to be a matter of quantity (the number of elements in a repeat sequence) rather than quality. On the other hand, signs such as primitive reflexes and mirror movements did not involve movement sequences and might have different significance. Chen et al. [11] made a further differentiation among these items and included the signs that are manifested by spurious movements in a time and place where it is not expected to occur, into "disinhibition".

Secondly, the prevalence rate of neurological abnormalities is not clearly studied. Studies have shown that neurological signs are subject to ethnic bias [7]. Boks et al. [4] reviewed 17 studies on the prevalence of specified neurological signs in schizophrenia and normal controls, all of them were from western-based sample. Information from Asian-based sample is scarce. Chen and Chan [10] made use of their limited data from a Chinese sample and demonstrated that there was an ethnic difference between Chinese and Caucasian healthy subjects in neurological soft signs of sensory integration. Caucasian subjects had higher sensory integration signs than their Chinese counterparts, after controlling for age and intelligence. Caucasian subjects also tended to exhibit higher motor coordination and disinhibition signs than Chinese subjects.

Moreover, the prevalence rate of neurological abnormalities may vary as a function of the criteria used to define normality and abnormality, and such criteria have been highly inconsistent among comparison subjects to determine the cutoff score for normality [26]. Ismaill et al. [19] adopted a comprehensive test of neurological abnormalities in a group of schizophrenic patients, their nonpsychotic siblings, and a group of normal comparison subjects. They found that patients and non-affected siblings did show a higher prevalence in neurological assessment. None of the comparison subjects scored higher than 6 on the neurological assessment scale, but a score of 7 or higher was given to 67% of patients and 19% of non-affected siblings. A good sensitivity and specificity of the neurological assessment scale was established. However, despite the full use of the hard and soft signs in Ismail et al. [19] study, they did not discriminate the differential sensitivity and specificity of hard signs from soft signs. Given the significance of neurological abnormalities in schizophrenia and the potential impact of ethnicity on its prevalence rate, the purpose of the present study was to provide additional information of neurological abnormalities from a Chinese sample. In particular, given the speculation that there is ethnic variation of neurological abnormalities owing to the level of obstetric care [16], we aimed to examine the prevalence and nature of neurological abnormalities in a group of patients with schizophrenia and normal control subjects with the use of a comprehensive neurological inventory. We also aimed to study the different sensitivity and specificity of specific domain scores including neurological hard signs, soft signs, and total neurological signs

## 2. Methods and materials

A cross-sectional design was adopted and a total of 250 (172 men, 78 women) patients with schizophrenia were recruited from 4 regional hospitals in Hong Kong Special Administrative Region (Castle Peak Hospital, Kwai Chung Hospital, Lai Chi Kok Hospital, and Queen Mary Hospital). The entry criterion of schizophrenic group was the diagnosis of schizophrenia in any three stages of illness: subacute, rehabilitation, and chronic. Research diagnosis was made by semi-structured interview schedules according to the DSM-III-R [1], and was further gained consensus from two experienced psychiatrists. Exclusion criteria were a history of organic illness involving central nervous system, substance and/or alcohol abuse, clinical evidence of mental retardation. Two hundred and thirty-nine patients were right handed, 4 were left handed, and 7 were mix-handed according to the Edinburgh Handedness Scale. The mean age was 40.9 years ($SD = 11.1$). The mean number of years of education was 8.2 years ($SD = 3.2$). The mean illness duration was 14.3 years ($SD = 9.5$). The mean antipsychotic dosage (chlorpromazine equivalence) was 914.15 mg/day ($SD = 889.68$). The mean dosage of anti-cholinergic was 3.33 mg of benzhexol ($SD = 3.36$).

Another 90 (31 men, 59 women) normal controls were recruited through a series of public education events. They were all screened by psychiatrists using a semi-structured interview. Potential subjects with a history of psychiatric illness, central nervous system diseases, substance and/or alcohol abuse, or query of mental retardation were excluded. The mean age was 38.7 years ($SD = 12.5$). The mean number of years of education was 8.8 years ($SD = 3$). There was no significant difference between schizophrenic patients and normal controls in current age and education level.

Neurological examination was performed by psychiatrists using the Cambridge Neurological Inventory (CNI) [11]. The CNI offered instructions for eliciting and rating a comprehensive range of neurological signs in 7 subscales. Three of these CNI subscales addressed soft signs (motor coordination, sensory integration, and disinhibition), whereas the remaining 4 subscales addressed hard signs (extrapyramidal signs, dyskinesia, catatonia, and pyramidal signs). Table 1 summarizes the items in each subscale. In the original scale, scoring was made according to standardized anchor points to indicate "normal" response (0), "equivocal response" (0.5), "abnormal" response (1) or "grossly abnormal" response (2). In the present study, items scores were further collapsed into either "absent" (covering normal or equivocal scores) or "present" (covering abnormal or grossly abnormal scores). Interrater reliability on the subscale scores were calculated for each of the subscales based on investigators' ratings on 15 independent cases. The intraclass correlation coefficient for the CNI was 0.85 for the total CNI score. The intraclass correlation coefficients for the subscales were as follows: motor coordination (0.91), sensory integration (0.82), disinhibtion (0.9), extrapyramidal signs (0.51), dyskinesia (0.95), catatonia (0.45), and pyramidal signs (0.69).

### *2.1. Data analysis*

Owing to the skewed distribution of subscales scores, group comparisons of the neurological signs for the two groups were done by the Mann-Whitney U test. Prevalence rate of individual items of the CNI was computed between the two groups in terms of chi-squares. Relative receiver operating curves (ROC) [9] were used to describe the relationship of the sensitivity and specificity of the assessment instrument, contrasting the patients' and normal control's scores for the 7 subscales, total soft signs, total hard signs, and total neurological signs.

Sensitivity in ROC analyses identifies subjects of a particular group membership who have been accurately classified as members of that group because their

Table 1
Items included in the 7 subscales of the Cambridge Neurological Inventory

| | | |
|---|---|---|
| Soft signs | Motor coordination | Finger-thumb opposition, finger-thumb tapping, dysdiadochokinesia, fist-edge-palm test, Ozeretski test |
| | Sensory integration | Extinction, finger agnosia, stereognosia, graphaesthesia, left-right disorientation |
| | Disinhibition | Blinking during saccadic eye movements, lateral head movement during saccadic eye movements, unilateral winking, mirror movements (during finger tapping and dysdiadochokinesia, the go-nogo test |
| Hard signs | Extrapyramidal | Glabellar sign, increased limb tone, decrease associated movements in walking, shuffling gait, arm-dropping test, tremor, neck rigidity |
| | Dyskinesia | Trunk-limb dyskinesia, orofacial dyskinesia |
| | Catatonia | Gait mannerism, Gegenhalten, Migehen, imposed posture, exaggerated spontaneous movements, abrupt smooth spontaneous movements, iterative spontaneous movements, other abnormal spontaneous movements, mutism, overactivity, underactivity, automatic obedience, abnormal behaviour, echopraxia, perseveration |
| | Pyramidal | Plantar reflexes, hyperreflexia, hyporeflexia, decreased power in extremities |

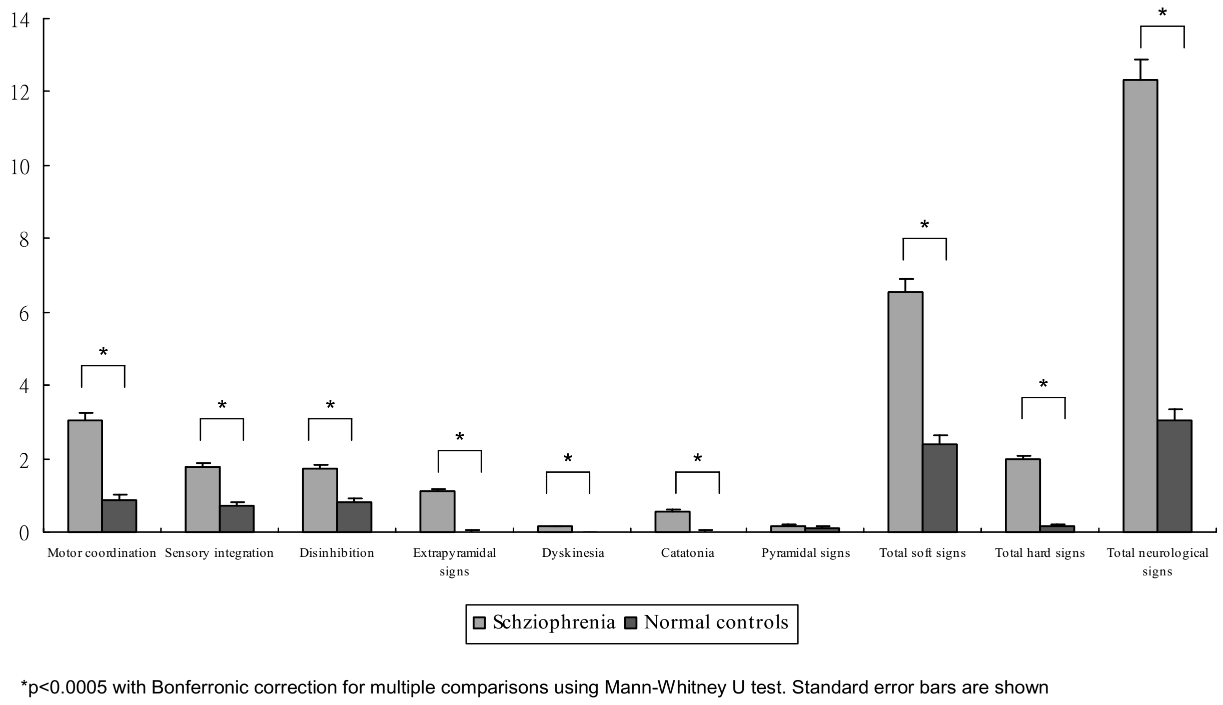

Fig. 1. Comparison of 7 subscales, total soft signs, total har d signs, and total neurological signs between schizophrenia and normal controls.

scores are above the selected cutoff score. Specificity is the proportion of subjects from another comparison group that is accurately classified as members of the comparison group because their scores fall below the selected cutoff score. ROC analyses plot the sensitivity and specificity of every possible cutoff score to obtain a curve that represents the distributional overlap between two groups on a given measure. By calculating the area under the curve (AUC) of the ROC, we can derive an index of the performance of a given measure, ranging from 0 to 1. An index of 1 indicates a perfect distinction between two groups, whereas an index of 0.5 indicates the two groups totally overlap with each other (represented by a 45-degree diagonal line on the ROC plot). The AUC value can then be interpreted as an estimate of the probability that a randomly chosen individual from one group will have a higher score on the measure than a randomly chosen individual from other group.

## 3. Results

### 3.1. Prevalence of neurological signs in schizophrenia and normal controls

Table 2 shows the prevalence of individual neurological items in patients with schizophrenia and normal

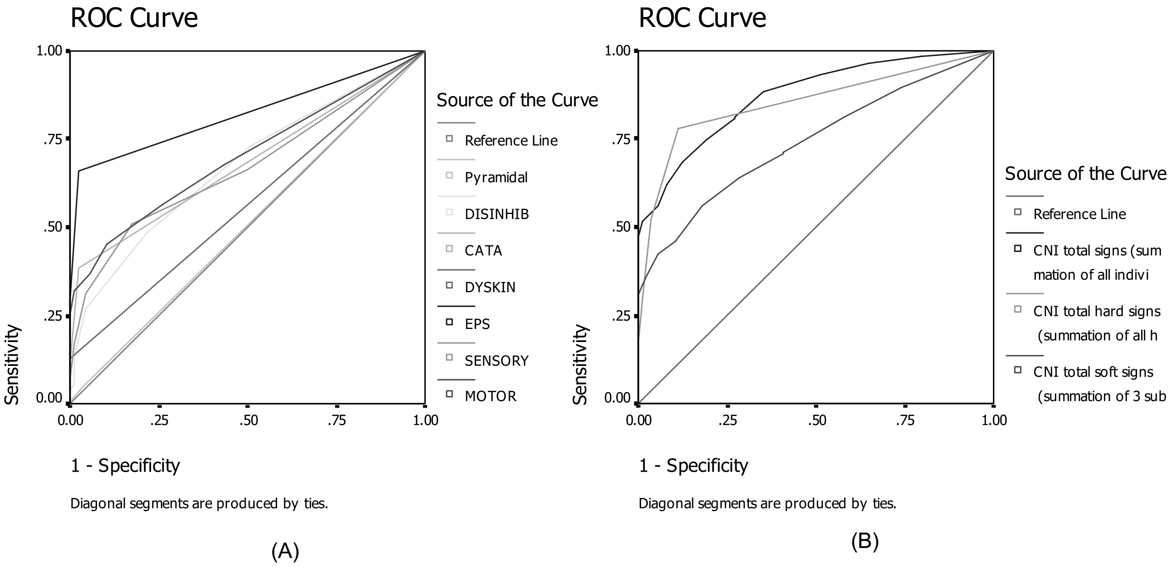

Motor=Motor coordination; SENSOR=Sensory integration; DISINHI=Disinhibition; EPS=Extrapyramidal; CATA=Catatonia; DYSKIN=Dyskinesia; Pyramid=Pyramidal signs;

Fig. 2. The ROC curves of the CNI subscales, total soft signs, total hard signs and total neurological signs.

controls. Significant differences were found between the two groups in most of the items, even after Bonferronic correction ($p < 0.0005$). A higher incidence of neurological signs was observed in schizophrenia than normal controls.

Figure 1 indicates that significant differences were found between patients and normal controls in all subscales but pyramidal signs ($p < 0.0005$). The difference between the patients and normal controls in the total abnormality and motor coordination scores remained significant even after filtering for extrapyramidal signs that may represent a medication effect ($p < 0.0005$).

### 3.2. *Sensitivity and specificity for different subscales of CNI*

Figure 2 shows the effect on the prevalence of neurological abnormality in the two groups when different cut-off scores for total neurological abnormality are used. At the extreme value of 100% specificity for schizophrenia, i.e. 0% of the comparison subjects with abnormality scores of $>= 6$ for motor coordination, neurological abnormality was found in 25.2% of patients. Furthermore, with a specificity of 90% for schizophrenia, i.e. fully 44.8% of patients were categorized as neurologically abnormal in motor coordination subscale of the CNI. Table 3 shows that the three subscales of soft signs, i.e. motor coordination, sensory integration and disinhibtion, get a relatively better sensitivity and specificity as compared with the remaining hard signs. The corresponding cut-offs for motor coordination is 2 (sensitivity $= 0.56$, specificity $= 0.73$), for sensory integration is 2 (sensitivity $= 0.5$, specificity $= 0.82$), for disinhibtion is 2 (sensitivity $= 0.48$, specificity $= 0.78$), for extrapyramidal signs is 1 (sensitivity $= 0.66$, specificity $= 0.98$), for dyskinesia is 1 (sensitivity $= 0.13$, specificity $= 1$), for catatonia is 1 (sensitivity $= 0.38$, specificity $= 0.97$), and for pyramidal signs is 1 (sensitivity $= 0.09$, specificity $= 0.92$), respectively.

When the subscales were collapsed into soft signs, hard signs and total signs, the sensitivity and specificity of the corresponding cut-offs were generally improved (Table 4). For the total soft signs, a cut-off of 4 yields a sensitivity of 0.63 and specificity of 0.71; whereas for the total hard signs, a cut-off of 1 yields a sensitivity of 0.78 and specificity of 0.89. A cut-off of 5 in the total neurological signs gets a sensitivity of 0.81 and specificity of 0.73.

## 4. Discussion

Results form this study provide additional information on the prevalence rate of neurological abnormalities from a Chinese sample. In general, schizophrenic

Table 2
Prevalence rate of individual items of neurological signs in schizophrenia and normal controls

| Signs | Schizoprehenia | | Normals | | Chi-Square | p-value |
|---|---|---|---|---|---|---|
| | $n = 250$ | % | $n = 90$ | % | | |
| Articulation | 42 | 16.80 | 0 | 0.00 | 17.25 | <0.0005 |
| Aprosody | 39 | 15.60 | 0 | 0.00 | 15.86 | <0.0005 |
| Unintelligible | 6 | 2.40 | 0 | 0.00 | 2.20 | ns |
| Extent SPEM | 23 | 9.20 | 1 | 1.11 | 6.60 | 0.007 |
| Smoothness SPEM | 86 | 34.40 | 11 | 12.22 | 15.96 | <0.0005 |
| Gaze Impersistence | 42 | 16.80 | 3 | 3.33 | 10.45 | 0.001 |
| Smoothness saccade | 70 | 28.00 | 4 | 4.44 | 21.56 | <0.0005 |
| Saccade blink | 92 | 36.80 | 12 | 13.33 | 17.16 | <0.0005 |
| Saccade head | 74 | 29.60 | 13 | 14.44 | 7.98 | 0.005 |
| Wink | 77 | 30.80 | 28 | 31.11 | 0.00 | ns |
| Glabeller sign | 56 | 22.40 | 1 | 1.11 | 21.49 | <0.0005 |
| Rapid tongue movement | 40 | 16.00 | 1 | 1.11 | 13.83 | <0.0005 |
| Impersistent tongue movt | 18 | 7.20 | 0 | 0.00 | 6.84 | 0.005 |
| Planter L | 2 | 0.80 | 0 | 0.00 | 0.72 | ns |
| Planter R | 2 | 0.80 | 0 | 0.00 | 0.72 | ns |
| Upper limb hypertonia | 19 | 7.60 | 0 | 0.00 | 7.25 | 0.005 |
| Upper limb inc str | 1 | 0.40 | 0 | 0.00 | 0.36 | ns |
| Upper limb hyperreflexia | 2 | 0.80 | 0 | 0.00 | 0.74 | ns |
| Upper limb hypotonia | 4 | 1.60 | 0 | 0.00 | 1.48 | ns |
| Upper limb weakness | 6 | 2.40 | 0 | 0.00 | 2.15 | ns |
| Upper limb hyporeflexia | 9 | 3.60 | 3 | 3.33 | 0.02 | ns |
| Lower limb hypertonia | 18 | 7.20 | 0 | 0.00 | 6.84 | 0.005 |
| Lower limb inc str | 0 | 0.00 | 0 | 0.00 | 0.00 | ns |
| Lower limb hyperreflexia | 5 | 2.00 | 2 | 2.22 | 0.01 | ns |
| Lower limb hypotonia | 2 | 0.80 | 0 | 0.00 | 0.72 | ns |
| Lower limb weakness | 0 | 0.00 | 0 | 0.00 | 0.00 | ns |
| Lower limb hyporeflexia | 10 | 4.00 | 5 | 5.56 | 0.34 | ns |
| Snout reflex | 0 | 0.00 | 0 | 0.00 | 0.00 | ns |
| Grasp reflex | 4 | 1.60 | 0 | 0.00 | 1.46 | ns |
| Palmomental relfex | 7 | 2.80 | 0 | 0.00 | 2.57 | ns |
| Finger-nose L | 15 | 6.00 | 0 | 0.00 | 5.65 | 0.01 |
| Finger-nose R | 18 | 7.20 | 0 | 0.00 | 6.84 | 0.005 |
| Finger-thumb tapping L | 47 | 18.80 | 1 | 1.11 | 17.08 | <0.0005 |
| Finger-thumb tapping R | 49 | 19.60 | 1 | 1.11 | 18.04 | <0.0005 |
| Finger-thumb Opposition L | 97 | 38.80 | 8 | 8.89 | 27.74 | <0.0005 |
| Finger-thumb Opposition R | 95 | 38.00 | 7 | 7.78 | 28.74 | <0.0005 |
| Mirror movement 1 l | 38 | 15.20 | 9 | 10.00 | 1.50 | ns |
| Mirror movement 1 R | 31 | 12.40 | 5 | 5.56 | 3.28 | ns |
| Dysdiadocokinesia L | 71 | 28.40 | 5 | 5.56 | 19.90 | <0.0005 |
| Dysdiadocokinesia R | 63 | 25.20 | 2 | 2.22 | 22.60 | <0.0005 |
| MIrror movement 2 L | 27 | 10.80 | 1 | 1.11 | 8.22 | 0.003 |
| Mirror movement 2 R | 36 | 14.40 | 2 | 2.22 | 9.89 | 0.001 |
| Fist-edge-palm L | 115 | 46.00 | 26 | 28.89 | 7.98 | 0.006 |
| Fist-edge-palm R | 116 | 46.40 | 14 | 15.56 | 26.66 | <0.0005 |
| Ozereski sign | 116 | 46.40 | 16 | 17.78 | 22.83 | <0.0005 |
| Rhythm | 147 | 58.80 | 20 | 22.22 | 35.43 | <0.0005 |
| Go-nogo | 53 | 21.20 | 2 | 2.22 | 17.58 | <0.0005 |
| Extinction | 15 | 6.00 | 0 | 0.00 | 5.65 | 0.01 |
| Finger angosia L | 103 | 41.20 | 25 | 27.78 | 5.08 | 0.03 |
| Finger angosia R | 105 | 42.00 | 23 | 25.56 | 7.62 | 0.007 |
| Stereognosis L | 16 | 6.40 | 0 | 0.00 | 6.44 | 0.04 |
| Stereognosis R | 20 | 8.00 | 0 | 0.00 | 8.06 | 0.01 |
| Graphesia L | 59 | 23.60 | 10 | 11.11 | 6.38 | 0.01 |
| Graphesia R | 57 | 22.80 | 4 | 4.44 | 15.15 | <0.0005 |
| Left right orientation | 67 | 26.80 | 4 | 4.44 | 20.02 | <0.0005 |
| Gait increased movement | 3 | 1.20 | 0 | 0.00 | 1.09 | ns |
| Gait decreased movement | 87 | 34.80 | 0 | 0.00 | 42.09 | <0.0005 |
| Gait shuffling | 26 | 10.40 | 0 | 0.00 | 10.14 | <0.0005 |
| Gait manneristic | 12 | 4.80 | 0 | 0.00 | 4.48 | 0.04 |

Table 2, continued

| Signs | Schizophrenia | | Normals | | Chi-Square | p-value |
|---|---|---|---|---|---|---|
| | $n = 250$ | % | $n = 90$ | % | | |
| Face dyskinesia | 14 | 5.60 | 0 | 0.00 | 5.26 | 0.025 |
| Face sustained | 20 | 8.00 | 0 | 0.00 | 7.65 | 0.003 |
| Face complex movement | 2 | 0.80 | 0 | 0.00 | 0.72 | ns |
| Gegenhalten | 5 | 2.00 | 0 | 0.00 | 1.83 | ns |
| Mitgehen | 5 | 2.00 | 0 | 0.00 | 1.83 | ns |
| Simple posture | 9 | 3.60 | 0 | 0.00 | 3.33 | ns |
| Complex posture | 2 | 0.80 | 0 | 0.00 | 0.72 | ns |
| Imposed posture | 0 | 0.00 | 0 | 0.00 | 0.00 | ns |
| Trunk-limb dyskinesia | 23 | 9.20 | 0 | 0.00 | 8.88 | 0.001 |
| Trunk-limb dystonia | 2 | 0.80 | 0 | 0.00 | 0.72 | ns |
| Trunk-limb mannerism | 29 | 11.60 | 0 | 0.00 | 11.41 | <0.0005 |
| Stand | 27 | 10.80 | 0 | 0.00 | 10.56 | <0.0005 |
| Arm drift | 27 | 10.80 | 1 | 1.11 | 8.22 | 0.003 |
| Arm drop | 3 | 1.20 | 0 | 0.00 | 2.65 | ns |
| Tremor | 38 | 15.20 | 0 | 0.00 | 14.40 | <0.0005 |
| Romberg sign | 8 | 3.20 | 0 | 0.00 | 2.95 | ns |
| Balance L | 33 | 13.20 | 1 | 1.11 | 10.75 | <0.0005 |
| Balance R | 33 | 13.20 | 1 | 1.11 | 10.75 | <0.0005 |
| Walk | 58 | 23.20 | 0 | 0.00 | 25.17 | <0.0005 |
| Tandem | 53 | 21.20 | 0 | 0.00 | 22.60 | <0.0005 |
| Abt sm | 1 | 0.40 | 0 | 0.00 | 0.36 | ns |
| Slow SM | 8 | 3.20 | 1 | 1.11 | 2.95 | ns |
| Iterative SM | 5 | 2.00 | 0 | 0.00 | 1.83 | ns |
| Ambivalence | 0 | 0.00 | 0 | 0.00 | 0.00 | ns |
| Mutism | 2 | 0.80 | 0 | 0.00 | 0.72 | ns |
| Neck rigidity | 10 | 4.00 | 0 | 0.00 | 3.71 | ns |
| Overactivity | 4 | 1.60 | 0 | 0.00 | 1.46 | ns |
| Underactivity | 29 | 11.60 | 0 | 0.00 | 11.41 | <0.0005 |
| Automatic obedience | 9 | 3.60 | 0 | 0.00 | 3.33 | ns |
| Noncompilance | 0 | 0.00 | 0 | 0.00 | 0.00 | ns |
| Abnormal behavoiur | 10 | 4.00 | 0 | 0.00 | 3.71 | ns |
| Echophenomenon | 48 | 19.20 | 3 | 3.33 | 13.07 | <0.0005 |
| Perseveration | 7 | 2.80 | 0 | 0.00 | 2.57 | ns |

patients exhibited a higher prevalence rate of neurological abnormalities than normal controls. When individual items were grouped into seven domain scores, all the soft signs domains and three out of four hard signs domains (except pyramidal signs) showed the same pattern of high scores in schizophrenic patients as compared with normal controls. This pattern persists even after filtering for signs that may represent a medication effect. These findings support the notion that, in general, patients with schizophrenia exhibit more neurological signs in individual items and domains of soft signs as well as hard signs.

Concerning sensitivity and specificity, satisfactory cut-offs of different subscales were established. Improvement in sensitivity and specificity was further demonstrated when the subscales were collapsed into total soft signs, total hard signs and total neurological signs. Unlike Ismail et al.'s [19] study, our findings show that similar discrimination of patients from normal subjects could be made with reference to total soft signs, total hard signs as well as total neurological signs, with a relatively superiority in classifying patients from normal controls using total neurological signs. Such a difference may be due to either the different items included in Ismail et al.'s study or differential prevalence rate of neurological abnormalities between Chinese and western samples. Items in the CNI do share major commonality with those used by Ismail et al.'s neurological examination. A minor deviation may not elevate such a significant difference to be observed in the sensitivity and specificity ranges. It seems that the assessment and the rating processes are not likely to be sources of variation. Therefore, it is more likely that ethnic variation in neurological abnormalities could be of the underlying source. Chen and Chan [10] and Gureje [16] suggested that such variation could be of a biological rather cultural nature. Gureje [16] speculated that increased neurological abnormalities in the Nigerian control subjects might relate to the level of obstetric care.

These findings also provide empirical support for the classification of soft signs into "motor coordination"

Table 3
Effect of different cutoff scores on prevalence of neurological abnormalities in schizophrenic patie and normal controls

| | Score | Schziophrenia | | Normals Controls | | Sensitivity | Specificity |
|---|---|---|---|---|---|---|---|
| | | Frequency | % | Frequency | % | | |
| Motor coordination | 0 | 81 | 32.40 | 51 | 56.67 | 0.32 | 0.43 |
| | 1 | 169 | 67.60 | 39 | 43.33 | 0.68 | 0.57 |
| | 2 | 140 | 56.00 | 24 | 26.67 | 0.56 | 0.73 |
| | 3 | 112 | 44.80 | 9 | 10.00 | 0.45 | 0.90 |
| | 4 | 91 | 36.40 | 5 | 5.56 | 0.36 | 0.94 |
| | 5 | 79 | 31.60 | 1 | 1.11 | 0.32 | 0.99 |
| | 6 | 63 | 25.20 | | | 0.25 | 1.00 |
| | 7 | 58 | 23.20 | | | 0.23 | 1.00 |
| | 8 | 31 | 12.40 | | | 0.12 | 1.00 |
| | 9 | 23 | 9.20 | | | 0.09 | 1.00 |
| Sensory integration | 0 | 84 | 33.60 | 45 | 50.00 | 0.34 | 0.50 |
| | 1 | 166 | 66.40 | 45 | 50.00 | 0.66 | 0.50 |
| | 2 | 124 | 49.60 | 16 | 17.78 | 0.50 | 0.82 |
| | 3 | 76 | 30.40 | 4 | 4.44 | 0.30 | 0.96 |
| | 4 | 41 | 16.40 | 1 | 1.11 | 0.16 | 0.99 |
| | 5 | 17 | 6.80 | | | 0.07 | 1.00 |
| | 6 | 11 | 4.40 | | | 0.04 | 1.00 |
| | 7 | 7 | 2.80 | | | 0.03 | 1.00 |
| | 8 | 1 | 0.40 | | | 0.00 | 1.00 |
| Disinhibtion | 0 | 69 | 27.71 | 44 | 48.89 | 0.28 | 0.51 |
| | 1 | 180 | 72.29 | 46 | 51.11 | 0.72 | 0.49 |
| | 2 | 119 | 47.79 | 20 | 22.22 | 0.48 | 0.78 |
| | 3 | 66 | 26.51 | 4 | 4.44 | 0.27 | 0.96 |
| | 4 | 32 | 12.85 | 1 | 1.11 | 0.13 | 0.99 |
| | 5 | 14 | 5.62 | | | 0.06 | 1.00 |
| | 6 | 9 | 3.61 | | | 0.04 | 1.00 |
| | 7 | 7 | 2.81 | | | 0.03 | 1.00 |
| Extrapyramidal signs | 0 | 84 | 33.60 | 88 | 97.78 | 0.34 | 0.02 |
| | 1 | 166 | 66.40 | 2 | 2.22 | 0.66 | 0.98 |
| | 2 | 72 | 28.80 | | | 0.29 | 1.00 |
| | 3 | 25 | 10.00 | | | 0.10 | 1.00 |
| | 4 | 11 | 4.40 | | | 0.04 | 1.00 |
| | 5 | 3 | 1.20 | | | 0.01 | 1.00 |
| | 6 | 1 | 0.40 | | | 0.00 | 1.00 |
| Dyskinesia | 0 | 218 | 87.20 | 90 | 100.00 | 0.87 | 0.00 |
| | 1 | 32 | 12.80 | | | 0.13 | 1.00 |
| | 2 | 5 | 2.00 | | | 0.02 | 1.00 |
| Catatonia | 0 | 154 | 61.60 | 87 | 96.67 | 0.62 | 0.03 |
| | 1 | 96 | 38.40 | 3 | 3.33 | 0.38 | 0.97 |
| | 2 | 27 | 10.80 | | | 0.11 | 1.00 |
| | 3 | 12 | 4.80 | | | 0.05 | 1.00 |
| | 4 | 4 | 1.60 | | | 0.02 | 1.00 |
| | 5 | 1 | 0.40 | | | 0.00 | 1.00 |
| Pyramidal signs | 0 | 223 | 90.65 | 81 | 92.05 | 0.91 | 0.08 |
| | 1 | 23 | 9.35 | 7 | 4.55 | 0.09 | 0.92 |
| | 2 | 12 | 4.88 | 3 | 3.41 | 0.05 | 0.97 |
| | 3 | 1 | 0.41 | | | 0.00 | 1.00 |

and "sensory integration" subgroups, similar to that proposed by Buchanan and Heinrichs [7]. The third subgroup, "disinhbition", is also valid in classifying patients from normal controls. In terms of sensitivity and specificity, "sensory integration" yields the best scores (cut-off: 2; sensitivity: 0.5; specificity: 0.82), followed by "motor coordination" (cut-off: 2; sensitivity: 0.56; specificity: 0.73), and "disinhibition" (cut-off: 2; sensitivity: 0.48; specificity: 0.78). Although we did not employ Buchanan and Heinrichs's [7] Neurological Evaluation Scale (NES) in our present study, we did check with and compute the estimated sensitivity and specificity indexes from the common items shared by the CNI. A cut-off of 1 in the NES "sensory integration" yields a sensitivity of 0.55 and a specificity of 0.81; a cut-off of 1 in the NES "motor coordination" yields a sensitivity of 0.48 and a specificity of 0.9; and a cut-off of 2 in the NES "sequencing of

Table 4
Effect of different cutoff scores on prevalence of soft signs, hards signs and total signs of CNI schizophrenic patients and normal controls

| | Scores | Schziophrenia | | Normals Controls | | Sensitivity | Specificity |
|---|---|---|---|---|---|---|---|
| | | Frequency | Cum % | Frequency | Cum % | | |
| Total soft signs | 0 | 27 | 10.84 | 23 | 25.56 | 0.11 | 0.74 |
| | 1 | 222 | 89.16 | 67 | 74.44 | 0.89 | 0.26 |
| | 2 | 201 | 80.72 | 53 | 58.89 | 0.81 | 0.41 |
| | 3 | 175 | 70.28 | 37 | 41.11 | 0.70 | 0.59 |
| | 4 | 157 | 63.05 | 26 | 28.89 | 0.63 | 0.71 |
| | 5 | 137 | 55.02 | 16 | 17.78 | 0.55 | 0.82 |
| | 6 | 113 | 45.38 | 9 | 10.00 | 0.45 | 0.90 |
| | 7 | 104 | 41.77 | 5 | 5.56 | 0.42 | 0.94 |
| | 8 | 89 | 35.74 | 2 | 2.22 | 0.36 | 0.98 |
| | 9 | 82 | 32.93 | 1 | 1.11 | 0.33 | 0.99 |
| | 10 | 75 | 30.12 | | | 0.30 | 1.00 |
| | 11 | 62 | 24.90 | | | 0.25 | 1.00 |
| | 12 | 50 | 20.08 | | | 0.20 | 1.00 |
| | 13 | 41 | 16.47 | | | 0.16 | 1.00 |
| | 14 | 34 | 13.65 | | | 0.14 | 1.00 |
| | 15 | 27 | 10.84 | | | 0.11 | 1.00 |
| | 16 | 21 | 8.43 | | | 0.08 | 1.00 |
| | 17 | 14 | 5.62 | | | 0.06 | 1.00 |
| | 18 | 10 | 4.02 | | | 0.04 | 1.00 |
| | 19 | 6 | 2.41 | | | 0.02 | 1.00 |
| | 20 | 4 | 1.61 | | | 0.02 | 1.00 |
| | 23 | 1 | 0.40 | | | 0.00 | 1.00 |
| Total hard signs | 0 | 54 | 21.95 | 78 | 88.64 | 0.22 | 0.11 |
| | 1 | 192 | 78.05 | 10 | 11.36 | 0.78 | 0.89 |
| | 2 | 129 | 52.44 | 3 | 3.41 | 0.52 | 0.97 |
| | 3 | 73 | 29.67 | 1 | 1.14 | 0.30 | 0.99 |
| | 4 | 41 | 16.67 | | | 0.17 | 1.00 |
| | 5 | 25 | 10.16 | | | 0.10 | 1.00 |
| | 6 | 14 | 5.69 | | | 0.06 | 1.00 |
| | 7 | 5 | 2.03 | | | 0.02 | 1.00 |
| | 10 | 2 | 0.81 | | | 0.01 | 1.00 |
| | 11 | 1 | 0.41 | | | 0.00 | 1.00 |
| Total signs | 0 | 4 | 1.60 | 18 | 20.45 | 0.02 | 0.80 |
| | 1 | 241 | 96.40 | 70 | 79.55 | 0.98 | 0.20 |
| | 2 | 236 | 94.40 | 57 | 64.77 | 0.96 | 0.35 |
| | 3 | 228 | 91.20 | 45 | 51.14 | 0.93 | 0.49 |
| | 4 | 217 | 86.80 | 31 | 35.23 | 0.89 | 0.65 |
| | 5 | 198 | 79.20 | 24 | 27.27 | 0.81 | 0.73 |
| | 6 | 183 | 73.20 | 17 | 19.32 | 0.75 | 0.81 |
| | 7 | 168 | 67.20 | 11 | 12.50 | 0.69 | 0.88 |
| | 8 | 152 | 60.80 | 7 | 7.95 | 0.62 | 0.92 |
| | 9 | 137 | 54.80 | 5 | 5.68 | 0.56 | 0.94 |
| | 10 | 126 | 50.40 | 1 | 1.14 | 0.51 | 0.99 |
| | 11 | 116 | 46.40 | | | 0.47 | 1.00 |
| | 12 | 106 | 42.40 | | | 0.43 | 1.00 |
| | 13 | 103 | 41.20 | | | 0.42 | 1.00 |
| | 14 | 99 | 39.60 | | | 0.40 | 1.00 |
| | 15 | 87 | 34.80 | | | 0.36 | 1.00 |
| | 16 | 79 | 31.60 | | | 0.32 | 1.00 |
| | 17 | 76 | 30.40 | | | 0.31 | 1.00 |
| | 18 | 64 | 25.60 | | | 0.26 | 1.00 |
| | 19 | 55 | 22.00 | | | 0.22 | 1.00 |
| | 20 | 45 | 18.00 | | | 0.18 | 1.00 |

complex act" yields a sensitivity of 0.54 and a specificity of 0.77. These indexes are comparable to those of ours, especially between CNI "disinhibition" and NES "sequencing of complex act".

The present study has a number of methodological limitations. The two groups were not matched for gender proportion. However, most previous studies did not show significant gender difference in neurological signs [12,18,37]. Lack of gender effect in neurological signs, particularly soft signs, would suggest that these aspects of the disorder are less likely to be involved in neurological signs expression.

The present sample recruited patients with relatively long period of illness duration and who were taking relatively large amounts of conventional antipsychotic medication. Most studies focusing on the relation between neurological signs and medication have argued against such a relation (e.g. [8,15,25]). Therefore, we cannot verify that the difference in prevalence rate of individual items and subscales of neurological signs was due to the medication side effect or not. However, after removing items that may be affected by medication, significant difference still remained between the two groups. Moreover, soft and hard neurological abnormalities are not only found in schizophrenic patients but also in patients with other mental disorders such as bipolar depression and ADHD. Therefore, these signs seem not to be specific for schizophrenia. Finally, the number of controls ($n = 90$) was rather small and not compatible to that of the schizophrenic group ($n = 250$). Future study recruiting more healthy controls and patients with first-episode schizophrenia should be conducted to further examine whether neurological signs have already increased in this disorder.

In conclusion, there is a high prevalence of neurological abnormalities in Chinese schizophrenic patients, comparable to that of the western cultures. An extended assessment battery of the Cambridge Neurological Inventory provides even better discrimination of patients from normal controls, and soft signs are similarly associated with schizophrenia than are hard signs in the Chinese sample.

## Acknowledgements

This study was supported partially by a Conference and Research Committee Grant and the Research Initiation Fund from the University of Hong Kong and the Institute of Psychology of Chinese Academy of Sciences. We thank patients and staff at Castle Peak Hospital, Kwai Chung Hospital, Lai Chi Kok Hospital, and Queen Mary Hospital for their participation and assistance in the study.
